# Specific absorption rate in neonates undergoing magnetic resonance procedures at 1.5 T and 3 T

**DOI:** 10.1002/nbm.3256

**Published:** 2015-01-16

**Authors:** Shaihan J. Malik, Arian Beqiri, Anthony N. Price, Jose Nuno Teixeira, Jeffrey W. Hand, Joseph V. Hajnal

**Affiliations:** ^1^ Centre for the Developing Brain and Department of Biomedical Engineering, Division of Imaging Sciences and Biomedical Engineering, King's College London, King's Health PartnersSt Thomas' HospitalLondonUK

**Keywords:** specific absorption rate, neonatal MRI, RF safety, electromagnetic simulations

## Abstract

MRI is finding increased clinical use in neonatal populations; the extent to which electromagnetic models used for quantification of specific absorption rate (SAR) by commercial MRI scanners accurately reflect this alternative scenario is unclear. This study investigates how SAR predictions relating to adults can be related to neonates under differing conditions when imaged using 1.5 T and 3 T MRI scanners. Electromagnetic simulations were produced in neonatal subjects of different sizes and positions within a generic MRI body transmit device operating at both 64 MHz and 128 MHz, corresponding to 1.5 T and 3 T MRI scanners, respectively. An adult model was also simulated, as was a spherical salt‐water phantom, which was also used in a calorimetry experiment. The SAR in neonatal subjects was found to be less than that experienced in an adult in all scenarios; however, the overestimation factor was variable. For example a 3 T body scan resulting in local 10 g SAR of 10.1 W kg^−1^ in an adult would deposit 2.6 W kg^−1^ in a neonate: an approximately fourfold difference. The SAR experienced by neonatal subjects undergoing MRI is lower than that in adults in equivalent situations. If the safety of such procedures is assessed using adult‐appropriate models then the result is a conservative estimate. © 2015 The Authors. *NMR in Biomedicine* published by John Wiley & Sons, Ltd.

**Abbreviations used**
SAR: specific absorption rate
SAR_10g_: local 10 g averaged SAR
TPN: total parenteral nutrition
IEC: International Electrotechnical Commission
ICNIRP: International Commission on Non‐ionizing Radiation Protection.

## Introduction

There is increasing interest in using MRI procedures on neonates 1. A major benefit of MRI is the absence of ionizing radiation, making it suitable for use on any patient group or on healthy volunteers. Nonetheless, safe operation of *in vivo* MR systems does require a strong safety culture and rigorous working practises compliant with strict safety guidelines. Careful control of power deposition from applied RF fields within the subject being examined is necessary to avoid temperature increases that are large enough to cause excessive physiological stress or pose a risk of tissue damage. Since it is not straightforward to directly monitor or accurately predict the spatial and temporal distributions of temperature in a subject, power deposition (as quantified by the specific absorption rate, SAR) is used as a surrogate. National and international regulatory authorities publish maximum limits on exposure in guidelines and standards 2, 3, 4. Table 1 lists temperature and SAR limits cited in the international standard IEC (International Electrotechnical Commission) 60601‐2‐33 2. Although the 2010 IEC 60601‐2‐33 standard contains limits for local SAR averaged over 10 g of tissue (SAR_10g_), these apply only in the case of *local* transmit coils, i.e. not for volume transmitters used in standard commercial whole body systems. As well as whole body and head averaged SAR, IEC 60601‐2‐33 also specifies limits for partial body SAR, which do apply for volume transmit coils. Partial body SAR is defined as SAR averaged over the exposed mass, and the limits specified vary depending on this mass as a fraction of the whole body mass; values quoted in Table 1 have been calculated for exposed mass fractions relevant to the scenarios studied in this paper. Details can be found in the Methods section. The International Commission on Non‐ionizing Radiation Protection (ICNIRP) statement on protection of patients undergoing MRI 3, which is still current regarding RF exposures 4, does impose limits on local SAR_10g_ in the head, trunk, and extremities, and it is common practice for research papers on SAR modelling for MRI to consider these limits. This is in part motivated by the fact that there are several examples in the literature of simulations of exposure to body coils that indicate that the SAR_10g_ limit is exceeded before the whole body SAR limit is reached 5, 6, 7, 8. All limits have been stated in Table 1, and adherence to all results in a conservative estimate of safety, but the reader should be aware of the differing standards.

**Table 1:** SAR and temperature limits within standard IEC‐60601‐2‐33

		Normal mode	First level controlled mode
Whole body SAR (W kg^−1^)		2.0	4.0
Head SAR (W kg^−1^)		3.2	3.2
Partial body SAR (W kg^−1^)	Adult exposure (43%)	6.6	7.4
	Baby head centred exposure (83%)	3.4	5.0
	Baby heart‐centred exposure (100%)	2.0	4.0
Local SAR_10g_ (W kg^−1^)	Head	10	20
	Trunk	10	20
	Extremities	20	40
Temperature (°C)	Maximum core temperature	39	40
	Max. local tissue temperature	39	40
	Increase in core temperature	0.5	1

SAR limits are averaged over 6 minutes; during any 10 s period SAR must not exceed twice the above values. For long duration examinations the specific absorbed energy must not exceed 240 W.minute kg^−1^ Operation in the normal mode does not cause physiological stress to patients; operation in the first level controlled mode can cause physiological stress to patients which needs to be controlled by medical supervision. According to IEC‐60601‐2‐33 (2010) the local SAR_10g_ limits only apply to local transmit coils (i.e. not the ones modelled in this study). Volume coils must however respect partial body SAR limits calculated from the exposed mass. For normal mode operation the Partial Body SAR limit is calculated as 10 W kg^−1^‐ (8 W kg^−1^ × *r*) and for the first controlled mode this is 10 W kg^−1^‐ (6 W kg^−1^ × *r*) where *r* is the exposed mass fraction (quoted as percentages in the table). Partial Body SAR limits for values of *r* relevant to the models in this paper are given.

Although it is possible to monitor the RF power produced by an MRI scanner, it is not possible to directly measure the resulting SAR distribution within the subject's body; to relate the two, safety checking algorithms employed by scanner manufacturers rely on predictions from computer models. The exact nature of these models is often undisclosed; however, in our experience they can include simple geometric shaped objects or human models. There is therefore some uncertainty in extrapolating the predicted values to neonatal subjects. Recent studies have considered multiple (adult and paediatric) models 9 and foetal subjects *in utero* 10, but none have considered directly the case of a neonate scanned *ex utero*. In this work we simulate exposure of a neonate to the RF field produced by generic birdcage coils at 1.5 T and 3 T using a digital anatomical phantom and a commercial electromagnetic solver. Two positions of the neonate relative to the coil (head and heart centred) are considered, as well as variants of the neonate voxel model with different physical sizes, with simulated touching heels and with insertion of a total parenteral nutrition (TPN) line. In each case the resulting whole body and head averaged SARs and local 10 g averaged SAR are predicted. An adult model is also included for the purposes of comparison with the neonatal models and with predictions from a commercial system's internal SAR model, whose details are unknown.

## Methods

### Numerical simulations

The Microwave Studio® Transient Solver within CST Studio Suite® 2013 (Computer Simulation Technology, Darmstadt, Germany) was used to solve the electromagnetic problem on personal computers with 3.6 GHz Intel® Core™ i7‐3820 processors and 16 GB RAM. Electric and magnetic field distributions at the operating frequency were extracted along with SAR distributions within the tissue models averaged over the head, the whole body), and locally over 10 g of tissue (SAR_10g_).

### RF transmit coil model

A generic birdcage transmit coil representative of the type used in commercial 1.5 T and 3 T MRI systems was modelled. Specifically this was a 16‐rung circular band‐pass birdcage coil with diameter 0.6 m and centre to centre spacing of the end rings 0.4 m, positioned within a 1.0 m long 1 mm thick cylindrical metal shield with internal diameter 0.678 m. The coil was tuned to 128 MHz (3 T) or 64 MHz (1.5 T) and driven in quadrature. All metallic components were assumed to have the same conductivity as copper (5.997 × 10^7^ S m^−1^).

#### Voxel models

Voxel models simulating neonates of term equivalent age (40 weeks gestational age) were created starting from an existing model of a deceased eight week old female baby of mass 4.2 kg and length 57 cm (24 h post mortem). The original model was derived from high resolution CT data segmented into 31 tissue types. It was acquired under a licensing agreement from the Institute of Radiation Protection (now Research Unit Medical Radiation Physics and Diagnostics) at the Helmholtz Zentrum München, German Research Centre for Environmental Health 11, 12. Since the original baby's weight was at the 97th percentile for term aged girls (95th percentile for boys) and its length exceeded the median by more than three standard deviations for both boys and girls 13, we scaled its voxels down by 10% in all three dimensions to create a simulated neonate (“Baby A”) of mass 3.02 kg (32nd percentile for females, 25th percentile for males at term equivalent age) and length 51.3 cm (88th percentile for females, 77th for males at term equivalent age) 13, resulting in 0.76 × 0.76 × 3.6 mm^3^ voxels. A second neonatal model more typical of female newborns was created by anisotropically scaling the original voxel model to produce “Baby B”, with 0.805 × 0.805 × 3.45 mm^3^ voxels resulting in mass 3.25 kg (52nd percentile for females, 42nd percentile for males) and length 49 cm (47th percentile for females, 32nd percentile for males).

**Figure 1:**
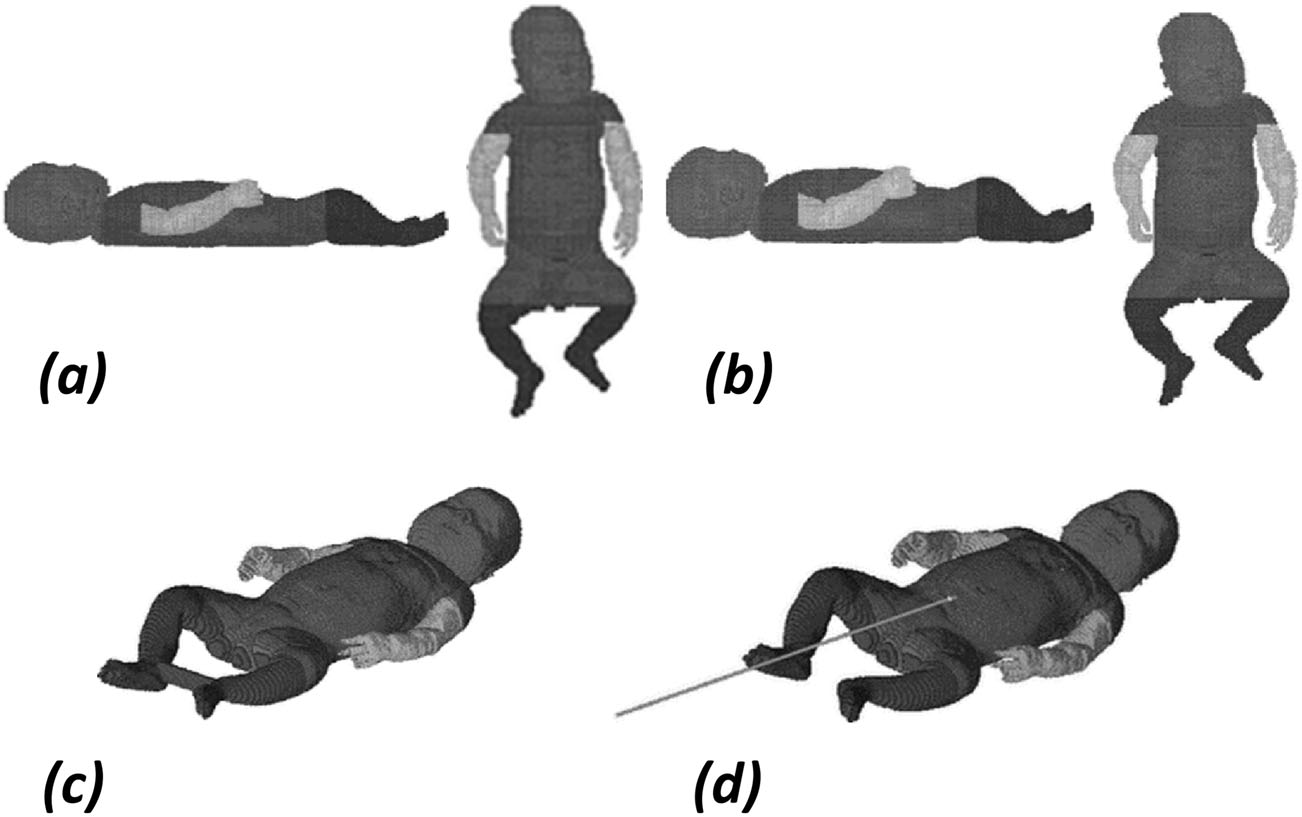
Baby models studied. (a) Baby A (lateral and anterior views), (b) Baby B (lateral and anterior views), (c) Baby A with heels connected, (d) Baby A with TPN line. (a) and (b), drawn to the same scale, indicate the differences in shape and size between Baby A and Baby B. The different grey shading indicates the segmentation of the limbs that was present in the original model.

Two further variants of the Baby A model were also investigated (Fig. 1). In one model the baby's heels were connected by a medium with permittivity of 80 and conductivity of 1 S m^−1^ (similar to 6 g L^−1^ saline at 20 °C 14) to simulate the effect of introducing a conducting loop, as might occur if the heels are touching or a saline bag is placed between them. In the second variant a baby undergoing TPN via a catheter with outer diameter 4 mm and wall thickness 1 mm inserted into the umbilical vein to a depth of approximately 50 mm via the umbilical stump was simulated. Externally to the baby, the catheter was modelled as oriented parallel to the axis of the transmit coil with length 0.55 m, taking it out of the RF coil. The major ionic constituents of the perfusate were taken to be sodium (6.25 mM), potassium (2.5 mM), calcium (1.9 mM), phosphate (2.5 mM), and chloride (3.8 mM). Permittivity and conductivity values of 80 and 0.2 S m^−1^ and 3.5 and 7 × 10^−17^ S m^−1^ were used for the perfusate and catheter, respectively. The properties of the perfusate would give the RF wavelength in this medium as approximately 0.26 m, and an approximate estimate of the *Q* factor of any resonance (estimated as the inverse of the loss tangent) is *Q* ≈ 3, suggesting that any resonant effect would be highly damped.

An adult male voxel model (NORMAN 15) of height 1.76 m and mass 73 kg with 2 × 2 × 2 mm^3^ voxels was also simulated, positioned with heart centred in the same coil. All models were simulated at 3 T; additionally, the Baby A heart‐centred model and the adult model were simulated at 1.5 T to provide direct comparisons at lower frequency.

### Dielectric properties

Although there is a considerable body of data relating to the permittivity and conductivity of adult tissues, there are fewer data describing the properties of neonatal tissues. Several studies have indicated that dielectric properties reflect age dependent changes in the water content of tissues 16, 17, 18. The dielectric properties required for the neonatal models used in this study were derived using a similar approach to that reported by Dimbylow *et al.* 19, who scaled adult dielectric properties by the ratios calculated between the dielectric properties of newborn and adult rats 18. In this work the relevant ratios are taken from Reference 18 for 130 MHz (the lowest frequency studied in Reference 18) and adult human values based on data from References 20, 21, 22 were obtained online 23; see Table 2 for all relevant data.

**Table 2:** Permittivity and conductivity of newborn tissues

Tissue	Permittivity ratio a	Newborn tissue Permittivity b		Conductivity ratio a	Newborn tissue Conductivity b (S m^−1^)		Density c (kg m^−3^)
		128 MHz	64 MHz		128 MHz	64 MHz	
Adrenals	1.28	85.50	94.65	1.7	1.3670	1.3231	1050
Bone	2.18	32.12	36.36	3.9	0.2623	0.2321	1562
Bladder wall	1.28	28.01	31.48	1.5	0.4468	0.4310	1050
Bladder contents	1.0	21.88	24.6	1.0	0.2979	0.2870	987
Brain	1.45	91.61	119.81	1.7	0.7883	0.6820	987
Breast	1.28	7.23	7.43	1.5	0.0454	0.0443	1050
Connective tissue	1.28	66.43	76.15	1.5	0.7470	0.7115	987
Eyes	1.28	83.30	96.38	1.5	1.3758	1.3240	1050
Eye lens	1.28	68.00	77.48	1.5	0.9129	0.8787	1050
Gall bladder wall	1.0	74.29	87.40	1.0	1.0409	0.9660	1050
Gall bladder contents	1.0	89.09	105.44	1.0	1.5753	1.4818	1050
Heart	1.28	108.09	136.33	1.5	1.1475	1.0176	1050
Kidney	1.34	120.41	158.87	1.5	1.2764	1.1120	1050
Large intestine wall	1.28	98.18	121.22	1.5	1.0560	0.9570	1050
Large intestine contents	1.0	63.60	72.20	1.0	0.7190	0.6880	987
Liver	1.14	73.40	91.84	1.3	0.6631	0.5824	1050
Lung	1.28	81.71	96.36	1.5	0.8627	0.7965	296
Muscle	1.39	88.36	100.41	1.7	1.2220	1.1699	1050
Oesophagus	1.28	95.97	109.85	1.5	1.3686	1.3167	1050
Ovaries	1.28	101.64	136.72	1.5	1.1836	1.0278	50
Pancreas	1.28	85.56	94.65	1.5	1.2057	1.1675	50
Skin	1.89	116.63	145.01	2.1	1.1413	1.0249	1105
Small intestine wall	1.28	112.91	151.50	1.5	2.5374	2.3871	1050
Small intestine contents	1.0	63.60	72.20	1.0	0.7190	0.6880	987
Spinal cord	1.28	56.52	70.48	1.5	0.5299	0.4683	1050
Spleen	1.14	94.75	126.04	1.5	1.0843	0.9671	1050
Stomach wall	1.28	95.97	109.85	1.5	1.3686	1.3167	1050
Stomach contents	1.0	63.60	72.20	1.0	0.7190	0.6880	987
Thymus	1.28	85.56	94.65	1.5	1.2057	1.1675	1050
Thyroid	1.28	85.56	94.65	1.5	1.2057	1.1675	1050
Uterus	1.28	96.72	117.91	1.5	1.4405	1.3659	987

a The permittivity (conductivity) ratio is (permittivity (conductivity) of newborn tissue)/( permittivity (conductivity) of adult tissue) at 130 MHz as reported by Peyman *et al.* 18.

b Newborn tissue permittivity (conductivity) values are the adult values (from References 20, 21, 22) multiplied by the appropriate ratio.

c Density values provided by Helmholtz Zentrum München.

### Partial body SAR calculation

Partial body SAR is calculated by averaging over the region of the body within the effective volume of the RF coil 2. This volume extends some way beyond the physical limits of the coil since incident fields decay through space. For the heart‐centred baby model the baby was fully within this volume (i.e. 100% exposure) while for the head centred baby this was 83% (exposed mass = 2.5 kg) and for the heart‐centred adult model the exposure was 43% (exposed mass = 31 kg). These exposure levels were used to calculate the resulting partial body SAR limits quoted in Table 1, and partial body SAR was calculated by numerically integrating the results over the specified volumes.

### Normalization of results

Simulations produce estimates of SAR and RF magnetic field strength (*B*_1_) for an arbitrary input power; generally, MRI scanners make *in situ* measurements of the NMR active circularly polarized component of *B*_1_ (referred to as *B*_1_^+^) and scale their output power accordingly. SAR values for *B*_1_^+^ = 1 μT at 100% duty cycle are quoted since these can be used to predict SAR for any sequence and MRI scanner with similar RF hardware by scaling appropriately. Normalization to 1 μT was achieved by dividing predicted SAR values by the square of the average predicted *B*_1_^+^ within a 50 mm diameter circular region of interest on the central transverse slice (thickness 5 mm) of the model. A small region was chosen in order to provide consistency over the multiple models of different shapes and sizes. Total accepted power – i.e. the amount of power dissipated by the coil into the subject and other loss mechanisms such as radiation and resistive loss – is also quoted for reference. Table 4 also quotes the SAR estimates scaled for actual *B*_1_^+^ and duty cycle conditions used by Philips 1.5 T (Achieva, software version R3.2.1) and 3 T (Achieva, software version R3.2.1) MRI scanners (Philips Medical Systems, Best, The Netherlands) set to scan at their maximum permissible SAR for normal operation. These scanners operate with their own models for estimating local and global SAR (details unknown) and quote the limiting value to the user. The limiting SAR quoted by the scanner for the 1.5 T example is whole body averaged SAR = 4 W kg^−1^. The 3 T system operates under two different regimes: (i) body scanning and (ii) head scanning. In the body scanning limit the scanner quotes local torso SAR_10g_ = 10 W kg^−1^, while for head scanning the limiting value is head averaged SAR = 3.2 W kg^−1^. The latter is more permissive, since it assumes an adult having a head scan, where most of the body is outside the scanner and therefore not exposed to the RF. The Philips scanner modelled in this work selects the head scanning SAR limit if a head receiver coil is used for signal reception. Neonatal subjects are routinely scanned using adult head coils for reception and therefore activate this model, irrespective of the fact that the neonate remains fully exposed to the RF field, hence both scenarios are evaluated. Scan protocols for all three of the above scenarios were defined for an adult subject of mass 73 kg, and the following conditions were then obtained from the user interface: for 1.5 T, *B*_1_^+^ = 23.0 μT, duty cycle = 3.9%; for 3 T “body limited”, *B*_1_^+^ = 13.5 μT, duty cycle = 1.4%; for 3 T “head limited”, *B*_1_^+^ = 13.5μT, duty cycle = 3.0%. Scaling of the SAR values was performed by multiplying the 1 μT 100% duty cycle values by (*B*_1_^+^/1 μT)^2^ × (duty cycle/100).

### Verification experiment

A calorimetry experiment was performed using a spherical phantom of internal diameter 100 mm filled with a salt‐water solution of concentration 6 g L^−1^, on a Philips 3 T Achieva MRI scanner. The phantom was wrapped in foam packing material and placed within two nested polystyrene boxes for thermal insulation. Temperature was recorded using a Luxtron FOT Lab Kit with two fibre‐optic temperature probes (Luxtron, now LumaSense Technologies, Santa Clara, CA, USA) located at the centre of the phantom (separation < 1 cm), and two additional probes measuring ambient air temperature in the scanner bore. Phantoms were left to temperature stabilize in the scanner room overnight before scanning, which was performed for 1 h with temperature recorded at 1 s intervals during the experiment and for an hour either side using TrueTemp v2.0 (Luxtron). SAR was calculated from $SAR=C\frac{\Delta T}{\Delta t}$, where *C* is the specific heat capacity of water (4118 J kg^−1^ K^−1^), Δ*T* is the measured temperature increase and Δ*t* is the scanning duration. SAR in the phantom was also modelled as described above using conductivity and relative permittivity estimated using relations from Reference 16 to be 0.9 S m^−1^ and 78 respectively. A map of the *B*_1_^+^ field was acquired using the actual flip angle imaging method 24 (repetition times 30 ms and 150 ms, flip angle 60°) as part of the phantom experiment, and simulated fields were normalized to match these.

## Results

### Verification experiment

Temperature readings were averaged across the two probes inserted into the phantom (these showed very similar trends but had a slight offset that was attributed to calibration differences). The temperature increased linearly during RF heating with a measured rate of change of 0.01280 ± 0.0005 °C min^−1^ obtained via linear regression (*R* ^2^ = 0.95), translating to a measured SAR of 0.88 ± 0.03 W kg^−1^. The high *R* ^2^ value indicates a strongly linear temperature increase, suggesting that other heat transfer mechanisms can be safely neglected. The temperature was not observed to change in the period after RF was switched off; linear regression gave the trend to be −0.0010 ± 0.0001 °C min^−1^ with *R* ^2^ = 0.04. The absence of prominent detectable temperature change in this period indicates that the phantom was well insulated and other heat transfer effects were not detected. The corresponding simulated whole phantom SAR was calculated by scaling the simulated SAR by the measured *B*_1_^+^ and correct duty cycle. For nominal *B*_1_^+^ = 1 μT, the true value was measured as *B*_1_^+^ = 1.08 ± 0.02 μT at the centre of the phantom, giving a prediction of 0.85 ± 0.04 W kg^−1^.

### SAR simulations

**Figure 2:**
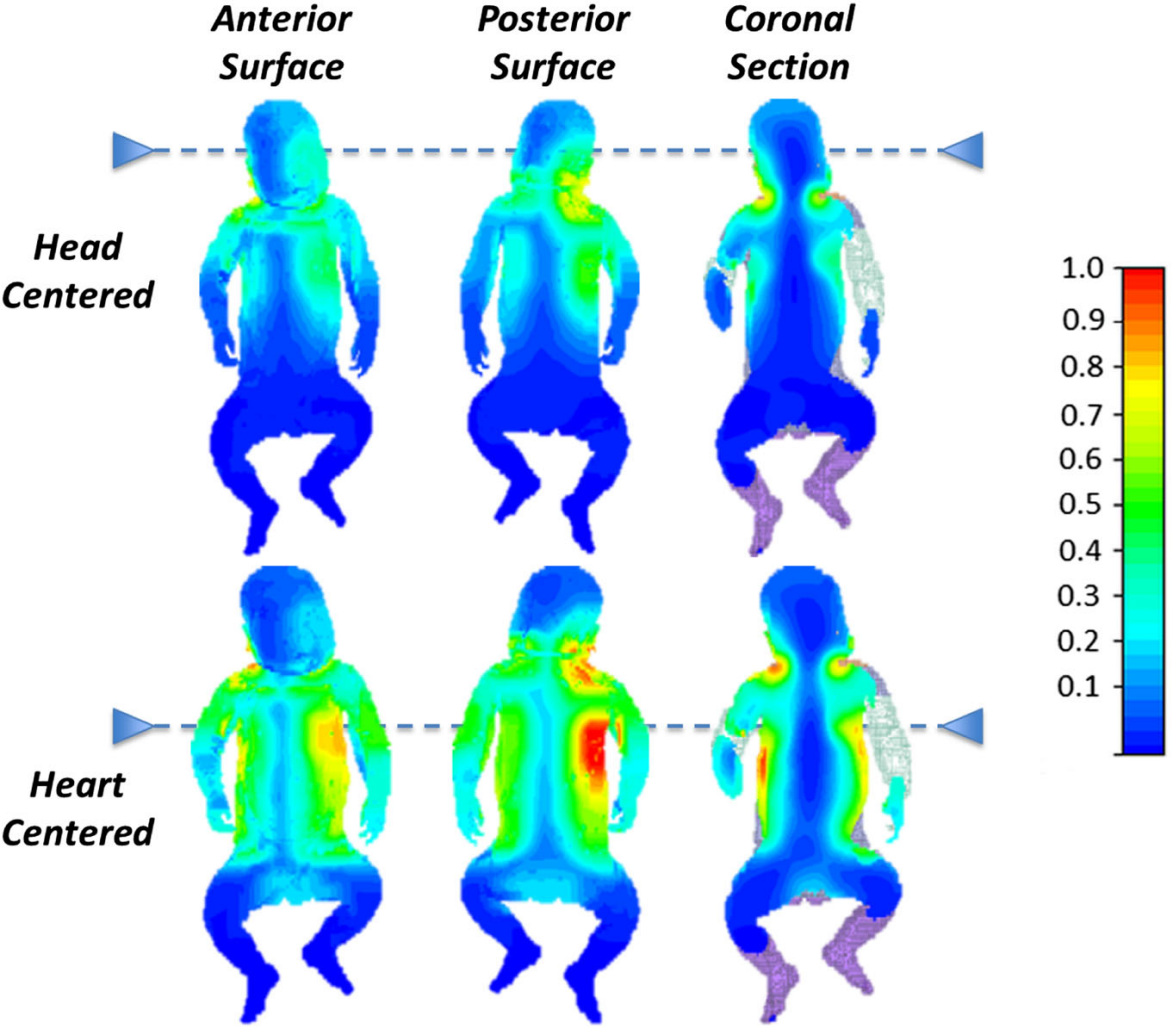
Local SAR_10g_ distributions on anterior surface (left), posterior surface (centre), and coronal section for (a) Baby A head centred and (b) Baby A heart centred. The dotted lines mark the centre of the RF coil in each case; all images have the same relative colour scale; for quantitative SAR measurements please refer to Tables 3 and 4.

Tables 3 and 4 summarize the numerical SAR predictions for 1 μT, duty cycle = 100%, and under realistic operating conditions respectively. Figure 2 shows some examples of spatial SAR distributions. In the case of the adult model, the predicted maximum local SAR_10g_ located in the trunk was 10.1 W kg^−1^ for a 3 T scan at the body scanning limit (*B*_1_^+^ = 13.5 μT, duty cycle = 1.4%); the Philips 3 T MRI scanner's estimate was 10 W kg^−1^. The same scan on Baby A yielded global SAR values ranging from 0.34 W kg^−1^ to 0.53 W kg^−1^, head averaged SAR from 0.37 W kg^−1^ to 0.55 W kg^−1^, and local SAR_10g_ from 2.15 W kg^−1^ to 2.53 W kg^−1^, depending on the specific model. For the heart‐centred baby model, since the baby is fully exposed to the RF fields, the partial body SAR and whole body SAR are the same. For the head centred baby (83% exposure) the partial body SAR was slightly more, at 0.4 W kg^−1^ (Table 4, first row). There is very little difference in the predictions for the heart‐centred models; the addition of a TPN feed line did not alter any of the quoted SAR values, and introducing a conductive medium between the heels changed values by less than 5%. Whole body averaged SAR is larger in the heart‐centred case while head averaged SAR is greater in the head‐centred case, as may be expected given the difference in position (Fig. 2; see index marks). Baby B is a more typically sized model for a newborn, and in this case too the results are similar to the heart‐centred Baby A models. If the scans were run at the adult head scanning limit (*B*_1_^+^ = 13.5 μT, duty cycle = 3.0%), the absolute SAR values increased, with whole body SAR ranging from 0.71 W kg^−1^ to 1.14 W kg^−1^ (including all models for both Baby A and Baby B), head averaged SAR from 0.77 W kg^−1^ to 1.15 W kg^−1^, and local SAR_10g_ from 4.48 W kg^−1^ to 5.40 W kg^−1^ (Table 4, right panel). At 1.5 T (*B*_1_^+^ = 23.0 μT, duty cycle = 3.9%) the predicted whole body SAR for baby A was 0.98 W kg^−1^, the head average was 0.65 W kg^−1^ and the local SAR_10g_ maximum was 4.38 W kg^−1^. For the adult model the values were higher, with partial body SAR of 3.7 W kg^−1^ and maximum SAR_10g_ of 27.2 W kg^−1^.

**Table 3:** Summary of simulated SAR results for 1 μT at 100% duty cycle

Model	Total accepted power (W)	Whole body averaged SAR (W kg^−1^)	Head averaged SAR (W kg^−1^)	Partial body SAR (W kg^−1^)	Max. local SAR_10g_ trunk (W kg^−1^)	Max. local SAR_10g_ head (W kg^−1^)
*127 MHz*						
Baby A head centred	0.85	0.13	0.21	0.15	0.82	0.88
Baby A heart centred	0.95	0.20	0.14	0.20	0.96	0.92
Baby A heart centred heels connected	0.95	0.20	0.14	0.20	0.93	0.89
Baby A heart centred TPN	0.95	0.20	0.14	0.20	0.96	0.92
Baby B heart centred	1.00	0.21	0.15	0.21	0.98	0.91
Adult heart centred	28.4	0.33	0.07	0.74	3.83	2.06
*64 MHz*						
Baby A heart centred	0.54	0.05	0.03	0.05	0.21	0.21
Adult heart centred	16.0	0.08	0.01	0.18	1.32	0.34

SAR simulation results, scaled for *B*_1_^+^ = 1 μT in a 50 mm region of interest close to the isocentre at 100% duty cycle.

**Table 4:** Simulated SAR results scaled to realistic operational values

	Scanner set at 100% body scanning limit					Scanner set at 100% head scanning limit				
Model	Whole body averaged SAR (W kg^−1^)	Head averaged SAR (W kg^−1^)	Partial body SAR (W kg^−1^)	Max. local SAR_10g_ trunk (W kg^−1^)	Max. local SAR_10g_ head (W kg^−1^)	Whole body averaged SAR (W kg^−1^)	Head averaged SAR (W kg^−1^)	Partial body SAR (W kg^−1^)	Max. local SAR_10g_ body (W kg^−1^)	Max. local SAR_10g_ head (W kg^−1^)
*127 MHz*										
Baby A head centred	0.34	0.55	0.40	2.15	2.31	0.71	1.15	0.84	4.48	4.82
Baby A heart centred	0.52	0.37	0.52	2.53	2.43	1.09	0.77	1.09	5.29	5.07
Baby A heart centred heels connected	0.53	0.37	0.53	2.45	2.35	1.10	0.77	1.10	5.11	4.91
Baby A heart centred TPN	0.52	0.37	0.52	2.53	2.43	1.09	0.77	1.09	5.29	5.07
Baby B heart centred	0.55	0.40	0.55	2.59	2.40	1.14	0.84	1.14	5.40	5.00
Adult heart centred	0.87	0.19	1.95	10.10	5.44	–	–	–	–	–
*64 MHz*										
Baby A heart centred	0.98	0.65	0.98	4.38	4.35	–	–	–	–	–
Adult heart centred	1.63	0.11	3.74	27.19	6.88	–	–	–	–	–

SAR values from Table 3 are here scaled to match operational limits from Philips 3 T and 1.5 T MRI systems. For body limited scanning, at 1.5 T *B*_1_^+^ = 23.0 μT, duty cycle = 3.9%; at 3 T *B*_1_^+^ = 13.5 μT, duty cycle = 1.4%. For 3 T head limited scanning, *B*_1_^+^ = 13.5 μT, duty cycle = 3.0%. The limits on SAR exposure are given in Table 1. Figures are not quoted for the head scanning limit for the adult model because it was simulated in a heart‐centred position, incompatible with a head coil, nor for the 1.5 T models as no such mode exists for 1.5 T scanning.

## Discussion

This study assessed the RF exposure of neonates undergoing MRI procedures. The algorithms implemented on commercial MRI systems to monitor RF power deposition are typically designed based on data from electromagnetic models whose exact nature is not disclosed, hence it is reasonable to assume that these may not necessarily provide accurate estimates for neonates. It was found for the neonatal models tested in this study that predicted SAR values were substantially lower than those obtained for a widely used model of an adult male under the same conditions. The closest result was the whole head average, which for the neonate was 57% of the adult value (head‐centred baby compared with heart‐centred adult both at 3 T) while the largest discrepancy was for maximum SAR_10g_ in body scanning conditions, where at 3 T the neonate experiences 23% of the adult value and at 1.5 T this dropped to only 16% of the adult value. Direct comparison of the results from the neonatal models simulated at both 3 T and 1.5 T showed that much lower SAR values were obtained than were reported by the commercial scanner software, indicating that the displayed SAR estimates were rather conservative. SAR calculations for the adult model agreed particularly well with the estimates provided by the commercial scanner software at 3 T, though no local SAR estimate was given by the scanner for 1.5 T. The calculation process was verified using calorimetry and found to give an accurate prediction to within experimental error.

The study considered different sized baby models and models with features designed to provoke potentially unsafe conditions (a TPN feed line and a saline bag introduced between the heels), but these did not result in major differences. A larger potential source of error when scanning at 3 T was found to be the use by the manufacturer of “head” and “body” limited scanning regimes, distinguished by the presence or absence of a head receiver coil. In the body limited case, a scan producing whole body SAR 0.87 W kg^−1^ and maximum local SAR_10g_ of 10.1 W kg^−1^ in an adult model produced whole body SAR 0.55 W kg^−1^ and maximum SAR_10g_ of 2.59 W kg^−1^ in a neonatal model; the local SAR for the neonate is just 26% of the recommended limit for normal operation 2, 3. A neonate positioned identically but scanned in the head limited regime would experience whole body SAR of 1.14 W kg^−1^ and maximum SAR_10g_ of 5.4 W kg^−1^. This approximately twofold increase in local SAR would not be apparent to the scan operator. Some caution is therefore advised when using adult head receivers for neonatal scans (arguably this is “off label” use); however, it should be stressed that the values obtained are still well within the IEC limits. Operators usually exercise a greater degree of caution with respect to SAR when scanning at 3 T than 1.5 T; however, this study found that whole body SAR was 0.98 W kg^−1^ and maximum SAR_10g_ was 4.38 W kg^−1^ in the neonate at 1.5 T, which are both approximately a factor of two higher than the body limited results at 3 T (but still within regulatory limits). Table 3 shows that when normalized to 1 μT the SAR predictions for 3 T are higher than 1.5 T by approximately a factor of 4, as would be expected. However the operational values in Table 4 are greater at 1.5 T than 3 T because the different scanners operate at different *B*_1_^+^ and duty cycles. The large margin of difference between the neonatal and adult models is however maintained at 1.5 T. As mentioned in the introduction, SAR_10g_ is not subject to control within the IEC standard for volume transmit coils; instead, partial body SAR is used. For a baby with 100% exposure the partial and whole body SAR values are the same, and the regulatory limits are also the same (see Table 1). For a head‐centred baby the partial body SAR is slightly higher, but the relevant regulatory limit is also increased; partial body SAR was not close to the stated limits in any of the scenarios investigated. Also note that the partial body SAR for the adult at 1.5 T was 3.7 W kg^−1^, well within the limit of 6.6 W kg^−1^ given in Table 1 for normal operation.

The clear finding of this study is that that in every scenario tested the SAR estimates produced by the MRI scanner were conservative for neonates. This may be seen as an intuitive result; indeed, simulated values for total accepted power also show that far more power is dissipated in the adult than the neonatal models: Table 3 shows accepted power is approximately 30 times greater for the adult models at both field strengths. These figures are approximately in line with predictions from scaling arguments such as those in Reference 25, which suggests that total losses in a spherical object scale with radius to the fifth power (a 30‐fold difference in power thus corresponds to approximately a twofold difference in radius, which is reasonable if comparing the adult and infant just in the axial plane). This type of argument cannot be used to justify safety on its own, since details of the overall field distributions, including local SAR_10g_ distributions, do not scale in this way. In view of these results it might be argued that neonates can be imaged safely using less stringent (more appropriate) RF exposure models. Since many rapid MRI protocols are limited by SAR concerns, use of neonate specific models could be used as a means of improving MR image quality in this population. However, such a change would only be appropriate if dedicated neonatal safety models are in place, as it is evident that while adult based models result in an increased safety margin they do not provide accurate estimates upon which to determine revised operating conditions.

Reliance on numerically predicted SAR must of course be placed in the context of uncertainties associated with the method of prediction. These can arise from numerous sources including numerical errors in the solver, uncertainty in the electrical model of the RF coil, normalization of the models, the small number of voxel models available to represent an overall population, and uncertainty in dielectric properties. In our own experience, when the subject can be modelled accurately, the numerical method is accurate to within less than 10% and usually better 26, 27. The phantom experiment in this study was one such scenario, and in this case achieved agreement with calorimetry to within experimental error. Since local SAR_10g_ is more dependent on local geometry than the global average, these values are subject to greater uncertainty, especially when attempting to represent a population using a small number of models. It is difficult to assess this uncertainty; a 30% estimate is given in the mobile communications literature 28 (in the GHz range); however, the lower frequencies used in MRI will lead to deviations closer to 10% 29. Our own SAR_10g_ estimate for an adult model at 3 T was very close to the scanner's prediction (10.1 W kg^−1^ compared with 10 W kg^−1^), though this may be coincidental given that the model used by the scanner's own software is unknown. In the case of neonates there is additional uncertainty due to the age dependency (through tissue water content) of dielectric properties and the changing composition of the subject, for example the amount of adipose tissue. The model used in this work did not contain a subcutaneous fat layer, instead only having a surface layer, which was treated as skin for the presented results. We found that if this was instead treated as a layer of pure fat the peak SAR_10g_ values were up to 10% lower, making our simulations a worst case. Taking all of these factors into account, we estimate that the overall uncertainty in SAR_10g_ if applying our results to a general neonate population is approximately 20%. Uncertainty in global average SAR is considerably less but is not the limiting case for safety.

Although SAR is commonly used in RF safety assessments, it is the combination of excessive temperature and its duration that has the potential to cause tissue damage. Indeed, local SAR_10g_ values have been shown to be only weakly associated with local tissue changes 30. Limits to tissue temperature and temperature change 2, 3 are listed in Table 1. These limits are based on the assumption that adverse health effects are not expected in people with unimpaired thermo‐regulatory and cardiovascular functions if the increase in body temperature does not exceed 1 °C 31. Thermal transport mechanisms in neonatal subjects differ from those in adults, however, and the ICNIRP standard 3 states that it is “desirable” to limit core temperature increases in infants to 0.5 °C. Neonates have a 2.5–3.0 times higher surface area to bodyweight ratio compared with adults, increasing the relative potential surface for heat loss 32. This is limited by the reduced insulating capacity from a lower quantity of subcutaneous fat and the inability of neonates to generate heat by shivering; the main concern for MRI of preterm neonates in this regard is actually keeping them warm. In contrast, the lower cerebral blood flow and immature peripheral circulation in neonates may have opposing effects on thermoregulation, as will swaddling or sedating infants in preparation for scanning. Such factors, along with the predicted SAR distributions, which indicate that the highest energy deposition is towards the surface of the neonate (Fig. 2), need to be considered in an appropriate thermal model to be investigated in the future.

In summary, this study provides evidence that SAR is lower in neonates than in adults under equivalent RF conditions (matched *B*_1_^+^ fields). SAR calculators designed for adults may therefore overestimate power deposition, creating an additional safety margin, but also potentially creating unnecessarily limiting operating conditions. However adjusting safety limits would not be wise without explicitly using dedicated neonatal models, since, as our calculations demonstrate, the margin of overestimation from adult based predictions is not consistent enough to act as the basis to estimate neonatal SAR.
